# Anti-Melanogenesis Effects of Lotus Seedpod In Vitro and In Vivo

**DOI:** 10.3390/nu12113535

**Published:** 2020-11-18

**Authors:** Jen-Ying Hsu, Hui-Hsuan Lin, Ting-Shuan Li, Chaio-Yun Tseng, Yueching Wong, Jing-Hsien Chen

**Affiliations:** 1 Department of Nutrition, Chung Shan Medical University, Taichung City 40201, Taiwan; jyhsu0530@gmail.com (J.-Y.H.); summer6279@yahoo.com.tw (T.-S.L.); winnielovejb3131@gmail.com (C.-Y.T.); wyc@csmu.edu.tw (Y.W.); 2 Department of Medical Laboratory and Biotechnology, Chung Shan Medical University, Taichung City 40201, Taiwan; linhh@csmu.edu.tw; 3 Department of Medical Research, Chung Shan Medical University Hospital, Taichung City 40201, Taiwan

**Keywords:** melanogenesis, α-MSH, UVB irradiation, lotus seedpod extract, epigallocatechin

## Abstract

Melanogenesis has many important physiological functions. However, abnormal melanin production causes various pigmentation disorders. Melanin synthesis is stimulated by α-melanocyte stimulating hormone (α-MSH) and ultraviolet (UV) irradiation. Lotus seedpod extract (LSE) has been reported as possessing antioxidative, anti-aging, and anticancer activities. The present study examined the effect of LSE on melanogenesis and the involved signaling pathways in vitro and in vivo. Results showed that non-cytotoxic doses of LSE and its main component epigallocatechin (EGC) reduced both tyrosinase activity and melanin production in the α-MSH-induced melanoma cells. Western blotting data revealed that LSE and EGC inhibited expressions of tyrosinase and tyrosinase-related protein 1 (TRP-1). Phosphorylation of p38 and protein kinase A (PKA) stimulated by α-MSH was efficiently blocked by LSE treatment. Furthermore, LSE suppressed the nuclear level of cAMP-response element binding protein (CREB) and disturbed the activation of melanocyte inducing transcription factor (MITF) in the α-MSH-stimulated B16F0 cells. The in vivo study revealed that LSE inhibited melanin production in the ear skin of C57BL/6 mice after exposure to UVB. These findings suggested that the anti-melanogenesis of LSE involved both PKA and p38 signaling pathways. LSE is a potent novo natural depigmenting agent for cosmetics or pharmaceutical applications.

## 1. Introduction

Melanin produced by melanocytes through melanogenesis directly affects the color of skin, hair, and eyes. Besides, melanin protects the skin from ultraviolet (UV) stimulation. However, abnormal melanogenesis causes hypo- or hyper-pigmentation disease and impacts the patient’s quality of life. Melanogenesis is influenced by intrinsic and extrinsic factors. Intrinsic factors of melanogenesis are resulted from overproduction of second messenger cyclic adenosine monophosphate (cAMP) which might occur during pregnancy and inflammation [1]. The major extrinsic factor of melanogenesis is UV radiation acting through the melanocortin-1 receptor (MC1R) that is activated by α-melanocyte-stimulating hormone (α-MSH) [2].

Melanogenesis is triggered by UV radiation or hormone stimulation such as α-MSH and adrenocorticotropic hormone (ACTH) or inflammation. Tyrosinase is the rate-limiting enzyme of melanin synthesis which is responsible for catalyzing L-tyrosine hydroxylation to levodopa (L-DOPA) [3]. Aside from tyrosinase, tyrosinase-related protein-1 (TRP-1) and tyrosinase-related protein-2 (TRP-2), resided in melanosome, also regulate melanin production [3]. The expressions of these enzymes were regulated by melanocyte inducing transcription factor (MITF), which has its specific DNA binding sequence to modulate melanocyte proliferation, differentiation, and melanogenesis [4]. The expression and activity of MITF were modulated by cAMP-responsive binding protein (CREB), mitogen-activated protein kinase (MAPK) family proteins, ribosomal S6 kinase (RSK), glycogen synthase kinase-3β (GSK3β), and p38 [5]. The binding of α-MSH to its receptor, MC1R, triggered intracellular cAMP levels, and induced cAMP-dependent signaling pathways [6]. It has been well established that cAMP elevation induced protein kinase A (PKA) activity and MAPK family proteins, including extracellular signal-regulated kinase (ERK), c-Jun N terminal kinase (JNK)1/2, and p38, were involved in melanogenesis signaling [6,7].

*Nelumbo nucifera* Gaertn., more commonly known as lotus, is a perennial hydrophyte which is mainly cultivated in high temperature and humidity climate patterns such as Taiwan, Japan, and some African countries. Most parts of lotus could be used as food or traditional Chinese medicine. However, lotus seedpod is a non-edible part of lotus and is usually discarded after harvest. Several studies have revealed that extract of lotus seedpods contained bioactive compounds. Proanthocyanin has been identified in lotus seedpods extract and was reported to exert antioxidative activity [8], antitumor activity [9], ameliorating cognitive impairment [10], inhibiting glycation [11], anti-inflammation [12], and anti-lipotoxicity [13]. According to the analysis of previous studies, EGC is abundant in LSE [12,14] that has been reported to have a depigmenting effect [15]. The present study used EGC as a reference standard to compare its efficacy with LSE.

The study is aimed to investigate the melanin synthesis effect and mechanism of LSE through in vivo and in vitro studies. Murine melanoma cells were used for the in vitro study under the induction of α-MSH. LSE or its main component EGC in various concentrations were applied to α-MSH-induced melanocyte to investigate the melanin synthesis effect and their possible mechanisms. Furthermore, LSE was applied topically to mice ears to confirm the melanin synthesis effect under repetitive UVB exposure.

## 2. Materials and Methods

### 2.1. Chemical and Reagents

α-MSH, EGC, H89, SB203580, and TRIzol reagent used in the present study were purchased from Sigma-Aldrich (St. Louis, MO, USA). Dulbecco’s modified Eagle’s Medium (DMEM), fetal bovine serum, penicillin/streptomycin, L-glutamine, and trypsin-EDTA (ethylenediaminetetraacetic acid) were purchased from Hyclone Laboratories (UT, USA). Antibodies including CREB, ERK (extracellular signal-regulated kinases), JNK1 (c-Jun N-terminal kinases 1), JNK2 (c-Jun N-terminal kinases 2), laminin B, MC1R, MITF, p-CREB, TRP-1, TRP-2, and tyrosinase were purchased from Santa Cruz Biotechnology (Dallas city, TX, USA). p38 MAPK, phospho-p38 (p-p38) MAPK, phospho-JNK, PKA, and p-PKA were purchased from Cell Signaling Technology (Beverly, MA, USA). Phospho-ERK and β-actin were purchased from Novus Biologicals (Littleton, CO, USA). The second antibody conjugated with horseradish peroxidase was purchased from Santa Cruz and Sigma-Aldrich.

### 2.2. Lotus Seedpod Extract (LSE) Preparation

The extraction of lotus seedpod was described in the previous study [12]. Briefly, dried lotus seedpods without lotus seeds were obtained from Baihe District, Tainan City, Taiwan. Dried lotus seedpods (100 g) were simmered in 4 L water at 95 °C for 2 h. After cooling, the aqueous extract of lotus seedpods was evaporated under vacuum at −80 °C. The decoction was concentrated and lyophilized as lotus seedpods extract powder (LSE) with a yield of about 18.4% from dried material. LSE powder was stored at −20 °C before use. The compounds of LSE have been identified and reported in the previous study [12].

### 2.3. Cell Culture

Murine melanoma cells B16F0 were purchased from Bioresource Collection and Research Center (Food Industry Research and Development Institute, Hsinchu City, Taiwan). B16F0 cells were cultured in DMEM supplemented with 10% FBS, 1% penicillin/streptomycin, and 1% glutamine at 37 °C in a 5% CO_2_ humidified incubator.

### 2.4. Cell Viability Assay

B16F0 cells were seeded in a 6-well plate (2 × 10^5^ cells/well) for 24 h. LSE was dissolved in deionized water with an equal volume of ethanol before use. EGC was prepared in deionized water. The cells were treated with/without LSE (5, 10, 15, 20, 25 μg/mL) or EGC (5, 10, 15, 20, 25 μM) for 48 h. Cell viability assay was analyzed by trypan blue, and live cells were measured and expressed as % of cell growth.

### 2.5. Tyrosinase Activity Assay

Tyrosinase activity assay was modified from the method described previously [16]. B16F0 cells were seeded in a 6-well plate (2 × 10^5^ cells/well) and treated with 1 μM α-MSH with/without LSE (10, 15, 20 μg/mL) and 15 μM EGC. After 48 h of incubation, cells were harvested by trypsin-EDTA and collected into a centrifuge tube. After being centrifuged, the cell pellet was washed with PBS twice and homogenized with PBS buffer containing 1% Triton X-100. Cell lysate (100 μL) and 2 μM L-DOPA in equal volume were mixed in a 96-well plate at 37 °C for 60 min. Tyrosinase activity in each treatment was determined at absorbance 490 nm with a SpectraMax M5/M5 Microplate Reader (Molecular Devices, Sunnyvale, CA, USA). Chemiluminescence (ECL) reagent was obtained from Millipore (Burlington, MA, USA). The tyrosinase activity of α-MSH-induced B16F0 cells with LSE, H89, and SB203580 treatment was assessed by the same method. The concentration of H89 or SB203580 was used according to the previous studies [17,18].

### 2.6. Melanin Content Assay

Melanin content assay was measured according to the method of Park et al. [19]. α-MSH-induced B16F0 cells were treated with/without LSE, EGC, H89, or SB 203580 for 48 h. The cell pellet was washed with PBS twice, and then 500 μL 1N NaOH was added and incubated at 80 °C for 1 h. Melanin content was measured at absorbance 409 nm with a microplate reader (SpectraMax M5/M5 Microplate Reader, Molecular Devices, Sunnyvale, CA, USA) and calculated from the melanin standard curve using synthetic melanin.

### 2.7. Cyclic AMP Assay

The intracellular cAMP level was measured by Cyclic AMP ELISA Kit (cat#581001, Cayman Chemical Company, Ann Arbor, MI, USA). According to the manufacturer’s instruction, cell lysate and cAMP-acetylcholinesterase (AChE) conjugate (cAMP tracer) were added and reacted in the pre-coated microplate. After washout with wash buffer, Ellman’s reagent, which contained the substrate of cAMP tracer, was added to wells and incubated for 90 min. The absorbance was measured at 412 nm with a microplate reader (SpectraMax M5/M5 Microplate Reader, Molecular Devices, Sunnyvale, CA, USA).

### 2.8. Real-Time PCR for Analyzing mRNA Levels

Primer sequences used in the present study were shown in Table 1. Total RNA was isolated from B16F0 cells by TRIzol reagent according to the manufacturer’s instructions. cDNA was synthesized from total RNA using the GoScript^TM^ Reverse Transcriptase System (Thermo Scientific, Rockford, IL, USA). The amplification of cDNA was performed using Power SYBR Green Master Mix (Applied Biosystems, Foster City, CA, USA) and detected by the StepOne™ Real-Time System using the comparative Ct (cycle threshold) method. Ct value in each sample was determined and normalized by the Ct value of *Gapdh* (∆Ct). The gene expression levels were calculated by the 2^-ΔΔCt^ method. Every experiment was conducted in triplicate.

### 2.9. Electrophoretic Mobility Shift Assay (EMSA)

Cell nucleus was isolated by the Mitochondria isolation kit for mammalian cells (cat#89874, Thermo Scientific, Rockford, IL, USA). The DNA-binding activity of CREB and MITF was assessed by EMSA. The sequences of DNA probe were listed in Table 2. Cell nuclear protein (10 μL) was mixed with 1 μL poly (dI-dC), 2 μL biotin-DNA probe, loading buffer, and binding buffer for the binding reaction. Protein and DNA complexes were separated by 8% negative-TBE (tris borate EDTA) PAGE (polyacrylamide gel electrophoresis) and transferred to a positive-charged nylon membrane. Crosslinking of the transferred membrane was performed under 1200 mJ/cm^2^ of UV light. The band shift was visualized by chemiluminescence and quantified via ImageQuant™ LAS 4000 mini (GE Healthcare Bio-Sciences AB, Uppsala, Sweden).

### 2.10. Animal and Experimental Design

Male C57BL/6 mice were purchased from the National Laboratory Animal Center (National Science Council, Taipei City, Taiwan). The animal procedure was approved and complied with the Chung Shan Medical University Animal Care Committee (IAUCC: 1255). Mice were housed in a constant humidity (55% ± 2%) and temperature (22 ± 2 °C) control room with a 12-h light/dark cycle. Food and drink (water) were given ad libitum during the experiment. Mice were randomly divided into five groups (*n* = 5) after adaptation for a week. The experimental groups were as follows: control group, UVB group, UVB + LSE 1.25 mg/cm^2^ group, UVB + LSE 2.5 mg/cm^2^ group, and UVB + EGC 2 mM/cm^2^ group. LSE was dissolved in an equal volume of water and alcohol and EGC in DMSO (dimethyl sulfoxide) before use. Mice ears were topically applied with LSE (1.25 mg/cm^2^ and 2.5 mg/cm^2^) or 2 mM/cm^2^ EGC before exposure to UVB. After about 30 min for the moisture to evaporate, mice were exposed to UVB irradiation (100 mJ/cm^2^). The UVB irradiation was conducted three times a week for 8 weeks and LSE or EGC was applied before UVB exposure. After 8 weeks of treatment, mice were sacrificed by carbon-dioxide asphyxiation followed by exsanguinations, and ear tissues were collected for melanin content and Western blot analysis.

### 2.11. Western Blotting

Mice ear skin tissue (1.5 g) was homogenized by RIPA buffer (radioimmunoprecipitation assay buffer) and centrifuged at 12,000× *g* for 10 min at 4 °C. The supernatant was transferred to a new microtube and stored at −80 °C before use. Protein concentrations in cell lysates or animal tissues were quantified by a Dual-Range™ BCA Protein Assay Kit (Energenesis Biomedical Co., LTD, Taipei city, Taiwan). Proteins (20–50 μg) were separated by SDS-PAGE and transferred to nitrocellulose paper. The membranes were blocked with 5% skim milk at 4 °C for 1–2 h before being incubated with primary antibody overnight at 4 °C. The appropriate horseradish peroxidase-conjugated secondary antibodies were incubated for 2 h at 4 °C. β-actin and laminin B were used as internal controls. Protein bands were visualized by enhanced chemiluminescence reagent. Protein levels were quantified by ImageQuant™ LAS 4000 mini (GE Healthcare Bio-Sciences AB, Uppsala, Sweden).

### 2.12. Statistical Analysis

Three or more separate experiments were performed. Data are reported as means ± standard deviation (SD) of three independent experiments. Student’s *t*-test was used for the analysis between two groups with only one factor involved. For the experiments of dose response of LSE or EGC, one-way analysis of variance (ANOVA) with post-hoc Dunnett’s test was used to calculate the *p*-value for each dose treatment compared to the α-MSH-treated group (α-MSH alone, without LSE or EGC), and regression was used to test the *p*-value of the dependency of a parameter to dosage. Significant differences were established at *p* < 0.05.

## 3. Results

### 3.1. Effect of LSE and EGC on Cell Viability in B16F0 Cells

**Figure 1:**
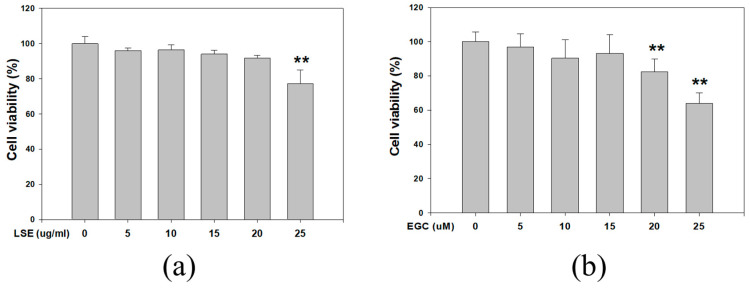
Effect of LSE or EGC on B16F0 cell viability. B16F0 cells were treated with various concentrations of (**a**) LSE (0, 5, 10, 15, 20, 25 μg/mL) and (**b**) EGC (0, 5, 10, 15, 20, 25 μM) for 48 h. The number of cells was counted by trypan blue dye exclusion assay. LSE: lotus seedpod extract. EGC: epigallocatechin. Values represent the mean ± SD of three independent experiments. ** *p* < 0.01, compared with control via one-way ANOVA with post-hoc Dunnett’s test.

The cell viability of B16F0 cells treated with different LSE and ECG concentrations is shown in Figure 1a,b. After being treated for 48 h, the survival rate was significantly decreased by 27% in 25 μg/mL LSE (Figure 1a). The survival rate of EGC treatment was significantly reduced by 36% in the 25 μM EGC group (Figure 1b). The concentrations of LSE below 20 μg/mL and EGC below 20 μM showed no cytotoxicity and were selected for investigating melanin formation.

### 3.2. Effect of LSE and EGC on Melanin Formation and Tyrosinase Activity in α-MSH-Induced B16F0 Cells

**Figure 2:**
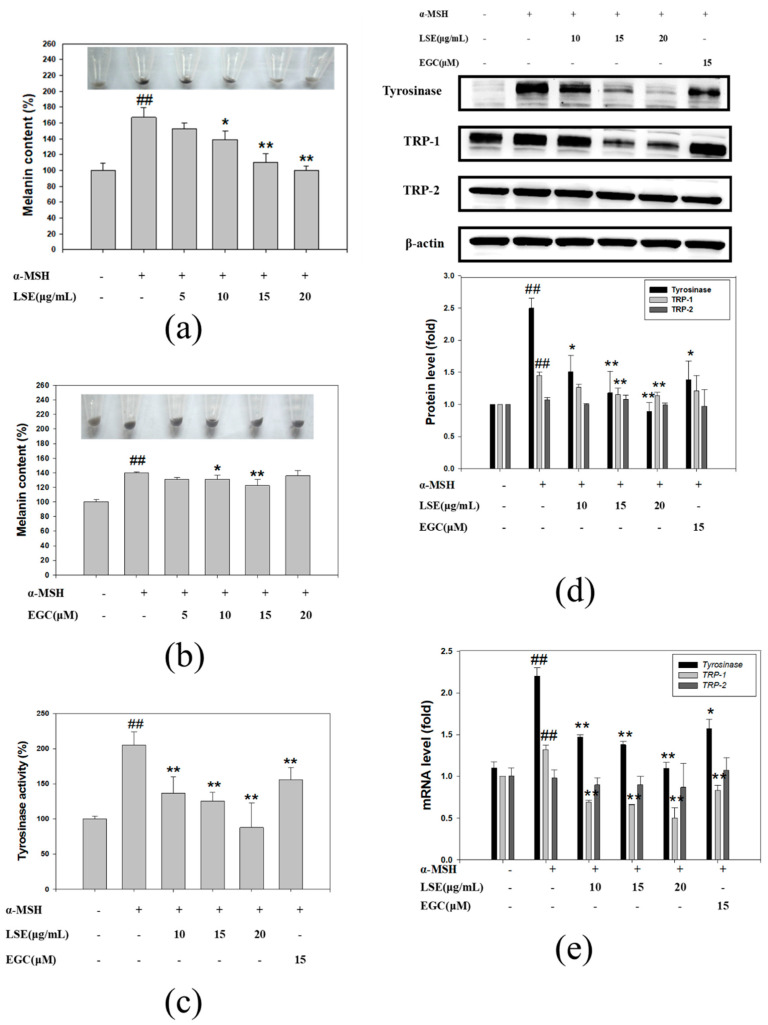
Effect of LSE or EGC on α-MSH-induced melanogenesis and tyrosinase activity in B16F0 melanoma cells. Cell pellets and melanin content of α-MSH-induced B16F0 cells treated with different concentrations of (**a**) LSE and (**b**) EGC. (**c**) Tyrosinase activity of B16F0 cells treated with LSE and EGC in various concentrations. (**d**) Tyrosinase, TRP-1, and TRP-2 proteins were analyzed by Western blotting. β-actin served as an internal control. (**e**) Tyrosinase, Trp-1, Trp-2 mRNA levels were analyzed by real-time PCR. α-MSH: α-melanocyte-stimulating hormone. LSE: lotus seedpod extract. EGC: epigallocatechin. TRP: tyrosinase-related protein. Values represent the mean ± SD of three independent experiments. ^#^^#^ *p* < 0.01 compared with control via student’s *t*-test. * *p* < 0.05, ** *p* < 0.01, compared with α-MSH-treated group via one-way ANOVA with post-hoc Dunnett’s test or via student’s *t*-test. +: added. -: non-added.

Melanin formation in the α-MSH-induced B16F0 cells treated with various LSE and EGC concentrations is revealed in Figure 2a,b. LSE of 10, 15, and 20 μg/mL doses significantly suppressed the melanin production in α-MSH-induced B16F0 cells, especially in 15 and 20 μg/mL LSE treatments whose cell pellets showed lighter colors (Figure 2a). Treatments of 10 μM and 15 μM EGC had significantly decreased melanin content when compared to the α-MSH group. Tyrosinase activities in LSE and EGC treatments were assessed and the results are shown in Figure 2b. According to the results, 10, 15, and 20 μg/mL LSE significantly inhibited 65%, 76%, and 112% tyrosinase activity in the α-MSH-induced B16F0 cells (Figure 2c). EGC treatment (15 μM) suppressed 47% of tyrosinase activity (Figure 2c). The results suggested that LSE and EGC reduced melanin production through repressing tyrosinase activity. Tyrosinase, TRP-1, and TRP-2 are important enzymes for catalyzing melanin formation in melanosome. The protein and mRNA levels in each group were analyzed and revealed in Figure 2d,e. Tyrosinase and TRP-1 protein and mRNA levels were significantly increased by α-MSH stimulation when compared with the control group, while TRP-2 protein and mRNA levels were similar to the control group. Tyrosinase protein and mRNA levels in 15 and 20 μg/mL LSE and 15 μM EGC treatments were significantly decreased in α-MSH-induced B16F0 cells. TRP-1 protein levels were significantly reduced by 66% and 69% by 15 and 20 μg/mL LSE (Figure 2d). LSE in 10, 15, and 20 μg/mL as well as 15 μM EGC treatments significantly inhibited TRP-1 mRNA levels by 196%, 207%, 256%, and 153% respectively, in α-MSH-induced B16F0 cells (Figure 2e). TRP-2 protein and mRNA expression remained unchanged after LSE and EGC treatments. Based on these results, LSE and EGC inhibited melanin formation through inhibiting tyrosinase and TRP-1.

### 3.3. Effect of LSE and EGC on cAMP/PKA Signaling Pathway and MAPK Pathway in α-MSH-Induced B16F0 Cells

**Figure 3:**
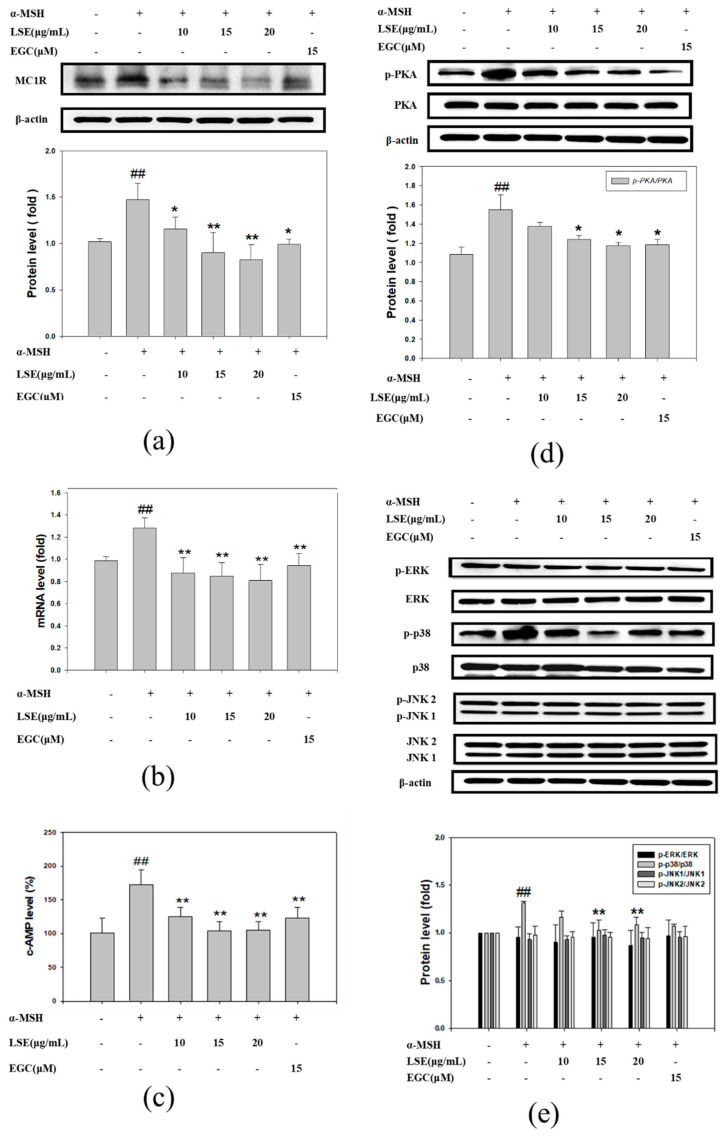
Effect of LSE and EGC on α-MSH-stimulated MC1R/cAMP/PKA signaling pathway in B16F0 cells. (**a**) MC1R protein and (**b**) Mc1r mRNA level in α-MSH-induced B16F0 cells treated with LSE or EGC in various concentrations. (**c**) Intracellular cAMP level was affected in LSE or EGC treatment. (**d**) p-PKA and PKA protein levels and (**e**) MAPK family proteins were analyzed by Western blotting. β-actin served as an internal control. α-MSH: α-melanocyte-stimulating hormone. LSE: lotus seedpod extract. EGC: epigallocatechin. MC1R: melanocortin-1 receptor. PKA: protein kinase A. ERK: extracellular signal-regulated kinases. JNK: c-Jun N-terminal kinases. cAMP: cyclic adenosine monophosphate. Values represent mean ± SD of three independent experiments. ^##^ *p* < 0.01 compared with control via student’s *t*-test. * *p* < 0.05, ** *p* < 0.01, compared with α-MSH-treated group via one-way ANOVA with post-hoc Dunnett’s test or via student’s *t*-test. +: added. -: non-added.

The MC1R protein and mRNA levels were investigated in LSE and EGC treatments. As shown in Figure 3a, α-MSH induced MC1R protein expressions but were markedly reduced by 126%, 142%, and 106% in 15 and 20 μg/mL LSE and 15 μM EGC treatments, respectively. MC1R gene expressions were analyzed by RT-PCR to evaluate the inhibitory effects of LSE and EGC. The results revealed that LSE and EGC treatments significantly repressed MC1R gene expressions by 143%, 153%, 168%, and 120% (Figure 3b). The binding of α-MSH to its receptor stimulated intracellular cAMP and PKA activation, and the downstream signal transduction of MC1R [20]. In agreement with the previous study, intracellular cAMP was significantly elevated by 70% in the α-MSH group when compared with the control group (Figure 3c). Treatments of 10, 15, and 20 μg/mL LSE markedly suppressed 66%, 95%, and 94% of cAMP level compared to that of the α-MSH group (Figure 3c). The cAMP level was decreased by 69% in the 15 μM EGC group. The ratio of phosphorylated PKA (p-PKA)/PKA was significantly elevated in the α-MSH group but reduced in 15 μg/mL and 20 μg/mL LSE and 15 μM EGC treatments (Figure 3d). In addition to the cAMP/PKA signaling pathway, the involvement of the MAPK pathway in the regulation was investigated by assessing ERK, p38, and JNK in α-MSH-induced B16F0 cells in the presence or absence of LSE and EGC. As shown in Figure 3e, p-ERK/ERK, p-JNK1/JNK1, and p-JNK2/JNK2 in the α-MSH-induced B16F0 cells revealed no change by LSE or EGC treatments. The p-p38/p38 level was increased by 31% in the α-MSH group but significantly reduced by 91% and 73% in 15 and 20 μg/mL LSE treatments. These data indicated that LSE treatment, especially in 15 and 20 μg/mL, suppressed melanogenesis in the α-MSH-induced B16F0 cells through regulating the cAMP/PKA signaling pathway and p-p38.

### 3.4. Effect of LSE and EGC on MITF and CREB in α-MSH-Induced B16F0 Cells

**Figure 4:**
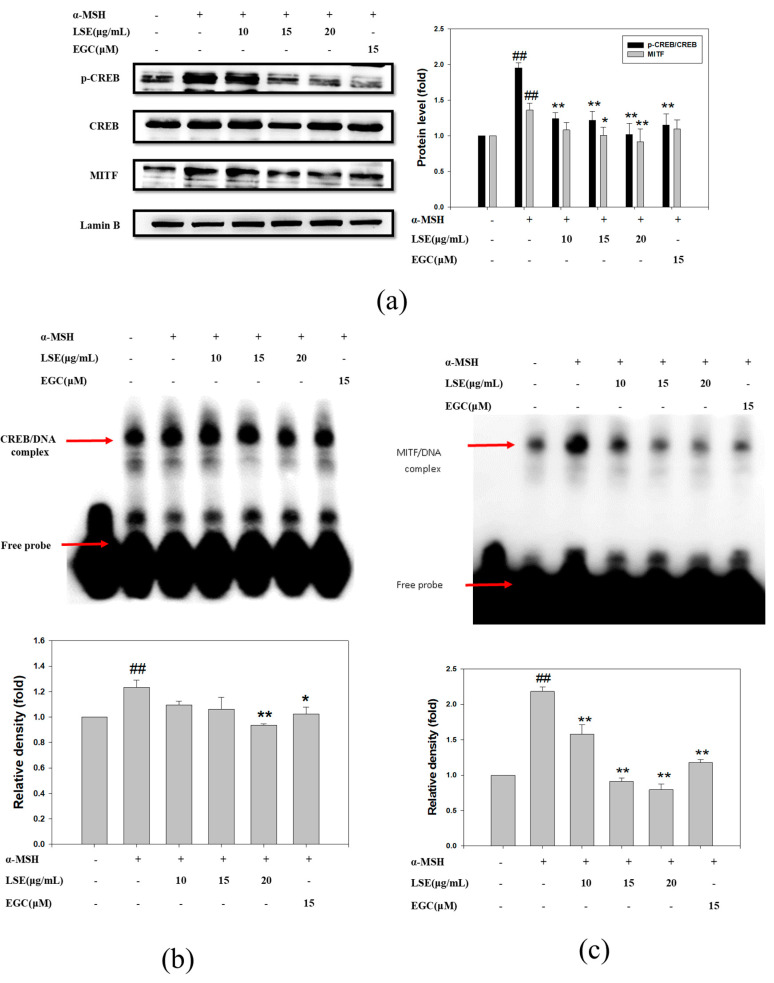
Effects of LSE on α-MSH-induced nuclear levels of p-CREB and MITF in B16F0 cells. (**a**) p-CREB/CREB and MITF levels were affected by LSE and EGC treatment, which was analyzed via Western blotting. Lamin B served as an internal control of nuclear fraction. (**b**) DNA-binding CREB activity of nucleus extracts which used biotin-labelled CREB-specific oligonucleotides by EMSA. (**c**) The nuclear extracts were analyzed for DNA-binding MITF activity of nucleus extracts, which used biotin-labelled MITF-specific oligonucleotides by EMSA. Lane 1 represents nuclear extracts incubated with unlabeled oligonucleotide (free probe) to confirm the specificity of binding. α-MSH: α-melanocyte-stimulating hormone. LSE: lotus seedpod extract. EGC: epigallocatechin. CREB: cAMP-response element binding protein. MITF: melanocyte inducing transcription factor. The results represent mean ± SD of three independent experiments and the significant difference was expressed as ^##^ *p* < 0.01 compared with control via student’s *t*-test. * *p* < 0.05, ** *p* < 0.01, compared with the α-MSH-treated group via one-way ANOVA with post-hoc Dunnett’s test or via student’s *t*-test. +: added. -: non-added.

Based on the results, LSE inhibited melanin synthesis through the cAMP/PKA pathway and p-p38. A previous study revealed that activated PKA phosphorylated CREB and MITF to promote melanogenesis gene expressions [21]. The present study analyzed CREB and MITF in the nucleus extracts of B16F0 cells. As shown in Figure 4a, the ratios of phosphorylated CREB (p-CREB)/CREB and MITF were significantly higher in the α-MSH-induced B16F0 cells than in the control group, while nucleus p-CREB/CREB levels were significantly reduced by 75%, 77%, and 98% in 10, 15, and 20 μg/mL LSE groups, and nucleus MITF levels were decreased in the LSE groups, especially or 15 μg/mL and 20 μg/mL, by 98% and 124%, as compared to the α-MSH group (Figure 4a). The EGC (15 μM)-treated group suppressed nucleus p-CREB/CREB protein levels but not MITF expression. To further confirm whether LSE or EGC treatment interfered with CREB or MITF binding to their promoters, EMSA was performed for analysis, and the results are shown in Figure 4b,c. The second lane in Figure 4b,c reveals that the biotin-labeled CREB or MITF probe was bound to the respective promoter and formed a DNA complex. According to the results, α-MSH stimulated CREB and MITF binding to the promoters 23% and 118% more than the control group (Figure 4b,c, lane 3). Compared with α-MSH (lane 3), 20 μg/mL LSE and 15 μM EGC significantly reduced CREB/DNA complex to 129% and 90%, respectively (Figure 4b, lanes 5 and 6). Besides, the MITF/DNA complex was significantly inhibited by 10, 15, and 20 μg/mL LSE and 15 μM EGC (Figure 4c, lanes 3 to 6). These results indicated that LSE and EGC interfered with the CREB and MITF binding to their promoters to regulate tyrosinase and *Tyrp1* gene expressions.

### 3.5. LSE Repressed Melanogenesis through Inhibiting PKA and p38 Signling Pathways

**Figure 5:**
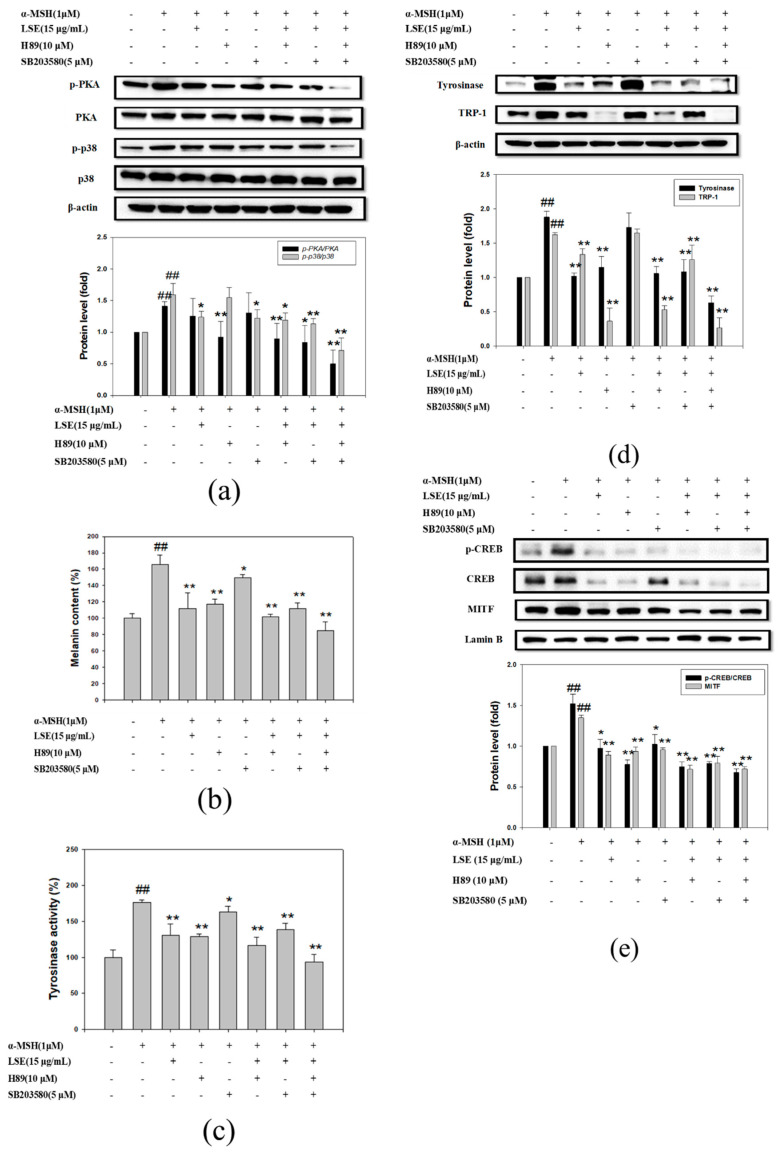
Effects of LSE, H89, and SB203580 on α-MSH-induced melanogenesis and tyrosinase in B16F0 cells. α-MSH-stimulated B16F0 cells were treated with 15 μg/mL of LSE or 10 μM of H89 (PKA inhibitor) or 5 μM of SB203580 (p38 inhibitor) for 48 h. (**a**) p-PKA, PKA, p-p38, and p38 protein levels were analyzed by Western blotting. β-actin served as an internal control. (**b**) Melanin content and (**c**) tyrosinase activity were determined in various treatments. (**d**) Tyrosinase and TRP-1 proteins were analyzed by Western blotting. β-actin served as an internal control. (**e**) The expression of p-CREB and MITF in the nucleus were determined by Western blotting. Lamin B served as an internal control of nuclear fraction. α-MSH: α-melanocyte-stimulating hormone. LSE: lotus seedpod extract. EGC: epigallocatechin. PKA: protein kinase A. TRP: tyrosinase-related protein. CREB: cAMP-response element binding protein. MITF: melanocyte inducing transcription factor. Values represent mean ± SD of three independent experiments. ^##^ *p* < 0.01 compared with control via student’s *t*-test. * *p* < 0.05, ** *p* < 0.01, compared with the α-MSH-treated group via student’s *t*-test. +: added. -: non-added.

To further confirm the involvement of PKA and p-p38 pathways in the melanogenesis inhibition of LSE, PKA inhibitor, H89, and p38 inhibitor, SB203580, were used for investigation. To begin with, 10 μM H89 or 5 μM SB203580 was treated along with α-MSH to confirm the inhibition of PKA and p38. As shown in Figure 5a, H89 and SB203580 significantly inhibited p-PKA/PKA and p-p38/p38 levels in the α-MSH-induced B16F0 cells. LSE alone reduced p-PKA/PKA to 38% and p-p38/p38 to 60% of those of α-MSH treatment (Figure 5a). LSE (15 μg/mL) along with H89 and SB203580 significantly repressed PKA and p38 activation by 221% and 148%, respectively (Figure 5a). Next, melanin content and tyrosinase activity in various treatments were shown, as in Figure 5b,c. The ability to reduce melanin content and tyrosinase activity by 15 μg/mL LSE was similar to H89 (Figure 5b,c). Tyrosinase and TRP-1 protein levels were significantly reduced by LSE with/without inhibitors in the α-MSH-induced B16F0 cells (Figure 5d). Nucleus extract of each treatment was analyzed to assess if LSE or inhibitors altered CREB and MITF expressions in the nucleus. The results are shown in Figure 5e: LSE alone or with inhibitors significantly decreased CREB phosphorylation and MITF levels in the α-MSH-induced B16F0 cells. According to these results, 15 μg/mL LSE inhibited melanin synthesis via repressing PKA and p38 and their downstream signaling pathways.

### 3.6. LSE and EGC Suppressed Melanin Synthesis Induced by UVB-Irridiation in Mice

**Figure 6:**
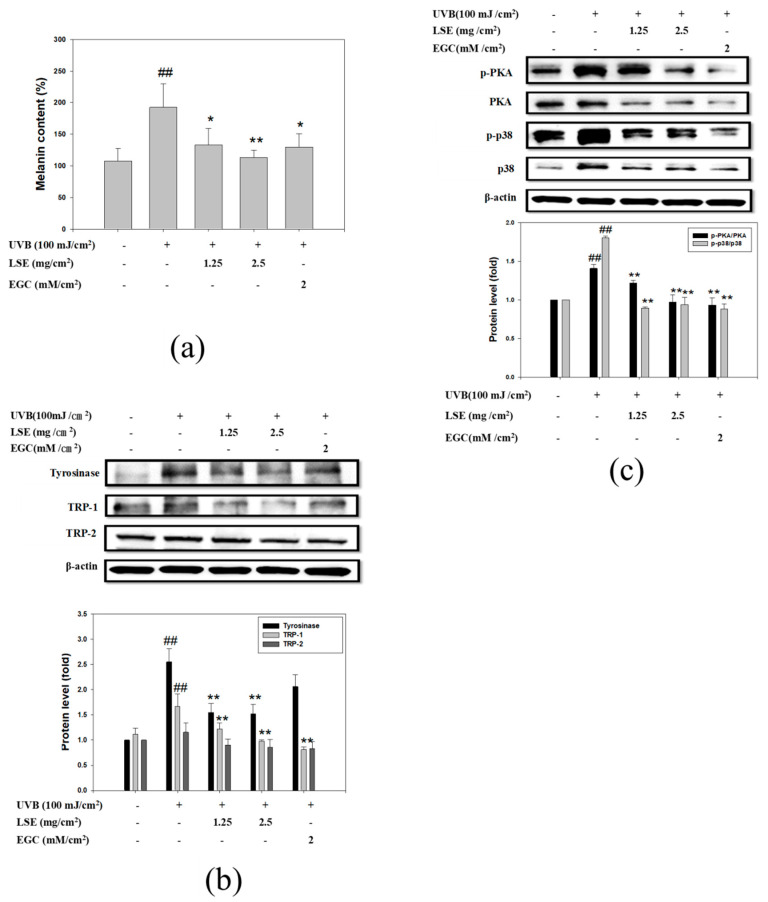
Effect of LSE and EGC on UVB-induced melanogenesis in mice ears. (**a**) Melanin content contained in mice ears with different treatments. (**b**) Tyrosinase, TRP-1, and TRP-2 proteins and (**c**) p-PKA, PKA, p-p38, and p38 proteins in ear tissues were analyzed by Western blotting. β-actin served as an internal control. UVB: ultraviolet radiation b. LSE: lotus seedpod extract. EGC: epigallocatechin. PKA: protein kinase A. TRP: tyrosinase-related protein. Values represent mean ± SD of three independent experiments. ^#^^#^ *p* < 0.01 compared with control via student’s *t*-test. * *p* < 0.05, ** *p* < 0.01, compared with UVB-treated group via one-way ANOVA with post-hoc Dunnett’s test or via student’s *t*-test. +: added. -: non-added.

According to the anti-melanogenesis mechanism of LSE and EGC in α-MSH-induced B16F0 cells, an animal study was designed to investigate the in vivo effect of LSE and EGC on melanin synthesis induced by UVB radiation. The dose of LSE applied on mouse ears was calculated by dividing 10 μg/mL, 20 μg/mL LSE, and 15 μM EGC treated to B16F0 cells by the culture dish area and to infer the amount of LSE from each cell exposed, then magnify it thousand times. Thus, the doses of LSE and EGC were 1.25 mg/cm^2^, 2.5 mg/cm^2^, and 2 mM/cm^2^, respectively. Mouse ear skin was analyzed by western blot to evaluate the molecular mechanism of LSE in melanin synthesis. Mouse ear samples were treated with 1 M NaOH at 80 °C for 1 h to dissolve melanin for content determination (Figure 6a). As shown in Figure 6a, melanin content in UVB irradiation treatment was significantly increased by 79% when compared with the control group. LSE of 1.25 mg/cm^2^ and 2.5 mg/cm^2^ treatments before UVB irradiation markedly reduced melanin content by 70% and 93% when compared with the UVB group (Figure 6a). Melanin content in 2 mM/cm^2^ EGC treatment was 74% lower than that of the UVB group. To determine the molecular pathway of melanin synthesis, tyrosinase, TRP-1 and 2, PKA, and p38 protein levels were analyzed by Western blot. Similar to the in vitro studies, when compared with the UVB group, tyrosinase and TRP-1 expressions in 1.25 mg/cm^2^ and 2.5 mg/cm^2^ LSE were significantly decreased, while there was no change in TRP-2 (Figure 6b). EGC (2 mM/cm^2^) revealed similar results with 2.5 mg/cm^2^ LSE treatment (Figure 6b). PKA and p38 phosphorylation were stimulated after UVB irradiation but were suppressed by 1.25 mg/cm^2^ and 2.5 mg/cm^2^ LSE as well as 2 mM/cm^2^ EGC treatment (Figure 6c). Consistent with the in vitro study, the in vivo study revealed that LSE and EGC treatment suppressed PKA and p38 phosphorylation that consequently reduced melanin synthesis after UVB irradiation.

## 4. Discussion

The present study investigated the anti-melanogenic effect of LSE intervention through in vitro and in vivo studies. It is the first study of LSE on melanogenesis. We investigated the non-toxic dose of LSE in B16F0 cells and selected 10, 15, and 20 μg/mL LSE, which reduced the melanin content without cytotoxicity. The possible anti-melanogenesis molecular pathways were analyzed in 15 and 20 μg/mL LSE and 15 μM EGC. We further confirmed the anti-melanogenetic effect of LSE via topical application to mouse ears. The results of both studies revealed that LSE reduced melanin synthesis by regulating CREB phosphorylation and MITF to inhibit tyrosinase and TRP-1 activity and expressions.

Our previous study analyzed LSE by liquid chromatography and found that its most abundant polyphenol was EGC [12]. The concentrations of LSE (10, 15, and 20 μg/mL) used in the present study were equivalent to 5.39, 7.1, and 8.9 μM EGC, respectively. The depigmenting effect of EGC in 10 and 20 μM has been reported to be significant in suppressing tyrosinase activity [15]. However, EGC 20 μM showed cytotoxicity when compared with the control group (Figure 1b). EGC 15 μM is equivalent to 25 μg/mL LSE, which revealed cytotoxicity to B16F0 cells. Hence, 15 μM EGC was used in the present study as a reference standard and we compared its efficacy on melanogenesis with LSE. According to the results, LSE and EGC treatment reduced melanin content in the α-MSH-induced B16F0 cells by suppressing tyrosinase activity and the expressions of tyrosinase and TRP-1, both of which were critical enzymes of melanin synthesis and were modulated by MC1R-induced downstream signaling pathways [21,22]. The binding of α-MSH to its receptor MC1R induced cAMP elevation, as the second messenger, to activate PKA [23]. Besides, p38 has been reported, whose activity was cAMP-dependent and regulated melanogenesis and proliferation of the α-MSH-induced melanoma cells [24]. Consistent with previous studies, the inhibition of melanin synthesis by LSE involved the cAMP/PKA signaling pathway and p38 phosphorylation (Figure 3) in the α-MSH-induced B16F0 cells. Activated PKA increased CREB phosphorylation that bound to its specific sequence to promote MITF expression [2]. MITF is an important transcription factor of melanogenesis including *Tyrosinase* and *Tyrp1* [23], proliferation, and differentiation [25]. The activation or degradation of MITF was concerned with the MAPK pathway [25]. The present study found that LSE reduced CREB phosphorylation and MITF levels in the nucleus, and whose DNA binding complexes were significantly decreased in the α-MSH-induced melanocyte (Figure 3). These results suggested that the inhibitory effects of LSE on cAMP/PKA signaling and p38 phosphorylation contributed to the downregulation of CREB and MITF activity binding to their promotor sites, that consequently decreased *Tyrosinase*, *Tyrp1*, and *Mc1r* expression levels and resulted in reducing melanin content. Compared with EGC, LSE in 15 and 20 μg/mL showed stronger inhibition effects in melanin production. This result indicates that there are still unknown compounds in LSE which are potent in depigmentation or synergistically working with EGC on melanin synthesis.

To investigate the potency of LSE under the inhibitor of PKA or p38 on the mechanism of melanin synthesis, 15 μg/mL LSE were combined with/without H89 and SB203580 for the treatments. Based on the results, the PKA inhibition effect of 15 μg/mL LSE was less than H89, while equivalent to SB203580 on p38 inhibition. The pPKA/PKA level was reduced in the combination of 15 μg/mL LSE and SB203580, which indicated that LSE could suppress both PKA and p38 activation (Figure 5a). Although 15 μg/mL LSE and H89 inhibited tyrosinase and TRP-1 expressions in the α-MSH-induced melanocyte, 15 μg/mL LSE was equivalent to H89 on suppressing tyrosinase but weaker than H89 on TRP-1 inhibition (Figure 5d). SB203580 revealed no effect on both but significantly reduced when treated with LSE (Figure 5d). The changes of tyrosinase and TRP-1 levels in different treatments were in a similar trend as the results in Figure 5b,c. The melanin synthesis inhibition of 15 μg/mL LSE was equivalent to H89 but was more potent than SB203580. The inhibition effect of 15 μg/mL LSE on CREB and MITF expressions in the nucleus revealed equivalent outcomes as H89 or SB203580 alone (Figure 5e). Besides, the combination of LSE and H89 or SB203580 showed a stronger inhibitory effect on CREB and MITF. Based on the experiment, the anti-melanogenesis effect of LSE was mainly through reducing PKA and p38 activity that consequently decreased CREB and MITF on regulating melanin synthesis-related gene expressions.

To further confirm the anti-melanogenesis potential of the LSE in vivo, the well-established animal model of UV-induced hyperpigmentation in the C57BL/6 mouse skin was utilized to analyze the effect of extract on melanin production and melanogenesis signaling. The process of melanogenesis, including melanin synthesis and distribution, not only constitutes a complex series of enzymatic and chemical reactions, but is also implicated in the cellular interaction of skin layers [7,26]. It has been reported that pigments formed in melanocyte melanosomes are then stored in the basal layer of epidermal cells, as well as in dermal macrophages, which become melanophores [27]. In agreement with these reports, the results of the in vivo experiments in this study showed that UVB induced melanin synthesis, whereas LSE or EGC treatments decreased melanin content in the mixture of epidermis and dermis of the mouse ear (Figure 6a). Furthermore, the key enzymes of melanogenesis signaling, tyrosinase and TRP-1, decreased in the 1.25 mg/cm^2^ and 2.5 mg/cm^2^ LSE treatments. The changes of tyrosinase and TRP-1 but not of TRP-2 in the LSE treatment were observed in both in vivo and in vitro studies. This is probably related to different functions of TRP-1 and TRP-2, although whose amino acid had high structural and sequence similarity with tyrosinase [28]. It has been reported that TRP-1 was involved in stabilizing and activating tyrosinase and melanosome during melanin synthesis [3]. The abnormal changes of TRP-1 led to pigmentation disorders such as Vitiligo and Oculocutaneous albinism [21]. TRP-2 was also required for melanin synthesis [3], but its biological function still needs to be clarified. It has been suggested that there is a requirement of TRP-1 for melanin synthesis. LSE suppressed both tyrosinase and TRP-1, which demonstrated that LSE effectively inhibited pigmentation. A previous study reported that UVB-induced melanogenesis was mediated by PKA and p38 pathways [29]. Based on the results, phosphorylated PKA and p38 were increased after repetitive UVB exposure, whereas both were reduced by 1.25 mg/cm^2^ and 2.5 mg/cm^2^ LSE treatment. The activity of PKA and p38 regulated CREB and MITF in the nucleus and thus affected pigmenting gene expressions. Consistent with the in vitro study, 1.25 mg/cm^2^ and 2.5 mg/cm^2^ LSE treatments suppressed melanogenesis by inhibiting PKA and p38 activation. Earlier studies have shown that the melanin production in melanocytes is influenced by keratinocytes and fibroblasts [30,31]. Primary melanocytes and keratinocytes have some drawbacks such as the limited proliferation capacity and the donor variability [31]. Primary murine melanocytes defective at the Mc1r locus have been historically difficult to culture in vitro [32]. Their poor growth has been attributed to faulty MSH responsiveness and decreased survival. Accordingly, current protocols for culturing primary murine melanocytes rely on pharmacologic manipulation [32]. Thus, the use of immortalized cell lines would be more suitable for investigating the effects of pigmentation regulators. B16 melanoma cells have been used widely in the pigmentation research model for normal melanocytes’ behavior. In addition, B16F0 cells were less malignant compared with other B16 melanoma sublines, suggesting that this clone was similar to normal melanocytes. Consistent with this is the demonstration by Bellei et al., showing a similar response of B16F0 and normal human melanocytes to promote melanogenesis when stimulating with various GSK3β-specific inhibitors [33]. Hwang et al. used the human epidermal melanocytes to explore the possible mechanism of maclurin, a natural xanthone, on melanin synthesis inhibition, including p38- and PKA-mediated signaling [34]. As the present study was a preliminary one to investigate the effects of LSE on melanogenesis, the effect of the extract upon normal melanocytes and UV irradiation on epidermal melanocytes separated from the dermis would be interesting to validate the photoprotective mechanism and thereby, needs to be explored in the future.

## 5. Conclusions

In summary, the present study first identified the melanin synthesis inhibition mechanism of LSE in the α-MSH-induced B16F0 cells through inhibiting PKA and p38 signaling pathways to repress melanin synthesis-related gene expressions. Consistent with the in vitro study, the inhibition effect of LSE was further confirmed in mouse ear skin. In conclusion, LSE possesses the potent efficacy of anti-melanogenesis and could be a novo depigmenting agent for cosmetics or pharmaceutical applications.

## Figures and Tables

**Table 1:** Primer sequence of the target genes for real-time PCR.

Gene	Sequence
*Tyrosinase*	Forward: 5′-CACCTGAGGGACCACTATTACG-3′
	Reverse: 5′-GGCAGTTCTATCCATTGATCCAG-3′
*Trp-1*	Forward: 5′-ATGAAATCTTACAACGTCCTCCC-3′
	Reverse: 5′-TGGCACACTCTCGTGGAAACTGA-3′
*Trp-2*	Forward: 5′-CTTCAACCGGACATGCAAATGC-3′
	Reverse: 5′-GCTTCTTCCGATTACAGTCGGG-3′
*Mc1r*	Forward: 5′-CTATGCGCTGCGTTATCACAG-3′
	Reverse: 5′-AAAGAAAGTGACGAGGCAGAG-3′
*Gapdh*	Forward: 5′-AGGTCGGTGTGAACGGATTTG-3′
	Reverse: 5′-GGGGTCGTTGATGGCAACAAT-3′

**Table 2:** Sequence of DNA probe.

Gene	Sequence
*CREB*	Forward: AGAGA TTGCC TGACG TCAGA GAGGT AG-5′ Biotin
	Reverse: CTAGC TCTCT GACGT CAGGC AATCT CT-5′ Biotin
*MITF*	Forward: ACTAC AACAC GTGTA GGCCA-5′ Biotin
	Reverse: TGGCC TACAC GTGTT GTAGT 5′-Biotin
